# Oncogenic and Tumor-Suppressive Functions of NOTCH Signaling in Glioma

**DOI:** 10.3390/cells9102304

**Published:** 2020-10-15

**Authors:** Elena Parmigiani, Verdon Taylor, Claudio Giachino

**Affiliations:** Department of Biomedicine, University of Basel, Mattenstrasse 28, 4058 Basel, Switzerland; elena.parmigiani@unibas.ch (E.P.); verdon.taylor@unibas.ch (V.T.)

**Keywords:** NOTCH signaling, glioma, glioblastoma, brain tumor, oncogene, tumor suppressor, neural stem cells, CSL, RBPJ, ASCL1

## Abstract

Although the role of NOTCH signaling has been extensively studied in health and disease, many questions still remain unresolved. Being crucial for tissue homeostasis, NOTCH signaling is also implicated in multiple cancers by either promoting or suppressing tumor development. In this review we illustrate the context-dependent role of NOTCH signaling during tumorigenesis with a particular focus on gliomas, the most frequent and aggressive brain tumors in adults. For a long time, NOTCH has been considered an oncogene in glioma mainly by virtue of its neural stem cell-promoting activity. However, the recent identification of NOTCH-inactivating mutations in some glioma patients has challenged this notion, prompting a re-examination of the function of NOTCH in brain tumor subtypes. We discuss recent findings that might help to reconcile the controversial role of NOTCH signaling in this disease, and pose outstanding questions that still remain to be addressed.

## 1. Context-Dependent Roles of NOTCH Signaling in Cancer

The NOTCH signaling pathway transduces short-range signals between adjacent cells and therefore is highly dependent on niche architecture. In mammals, signal activation is induced by direct interaction of one of the four NOTCH receptors (NOTCH1–4) with one of five canonical ligands (Jagged1, Jagged2, Delta-like 1 (DLL1), DLL3, DLL4), followed by two sequential proteolytic cleavages of the receptor by ADAM metalloproteases and the γ-secretase complex. Ligand-induced proteolysis of NOTCH receptors liberates the NOTCH intracellular domain (NICD). The canonical signal links the NICD to the nuclear CSL (CBF-1, Suppressor of Hairless, Lag-1; RBPJ in vertebrates) transcriptional regulator [1], which is part of a repressor complex that includes NCoR/SMRT and histone deacetylases, and is bound to the promoters of target genes. Upon NICD binding, the composition of the RBPJ complex changes and co-activators including mastermind-like protein 1 (MAML-1) and histone acetyltransferases (p300, CBP) are recruited to initiate gene transcription [2]. NOTCH controls the expression of a wide range of target genes on the basis of the tissue and cell type that reflects its multiple functions. The basic helix-loop-helix (bHLH) transcription factors of the HES/HEY family were the first NOTCH target genes to be described [3]. They mainly function as transcriptional repressors in heteromeric complexes with Transducin-Like Enhancer-of-split (TLE) proteins, and their expression is in some cases finely tuned by autoregulatory loops that result in oscillatory expression crucial to regulate, for example, stem cell maintenance and cell fate decision during embryonic development of the nervous system [4]. An additional level of regulation, at least in the brain, is exerted by a class of dominant negative regulators of bHLH proteins, the inhibitor of DNA-binding (ID) proteins. IDs can form heterodimers with bHLH factors and, in most cases, counteract their activity [5,6,7,8]. NICD/RBPJ can regulate the activity of gene promoters and play a fundamental role at distant long-range enhancers [9,10], as has been described in T cell acute lymphoblastic leukemia (T-ALL) [11,12].

NOTCH signaling is highly conserved from invertebrates to humans and is involved in the regulation of a variety of cellular processes throughout life, including cell proliferation, stem cell maintenance, cell fate decisions, and differentiation. Accordingly, deregulation of NOTCH signaling occurs in several human diseases, including cancer [1]. Due to its pleiotropic functions, NOTCH signaling is implicated in many aspects of tumor development where it can act either as an oncogene or a tumor suppressor, as extensively reviewed by others [13,14]. The balance between one or the other role is determined by many factors, such as the cell type, the stage of tumor development, or the target genes involved [13,14]. Genetic alterations in the genes of different NOTCH pathway components that can lead to either enhanced or decreased NOTCH signaling have been identified in multiple cancers [13,14,15]. Mutations that directly occur in NOTCH receptor genes can alter different portions of the protein [15]. NOTCH activating mutations often occur in the negative regulatory region (NRR), which regulates receptor cleavage, or in the PEST domain, which is involved in NICD degradation, thus leading to ligand-independent or prolonged receptor activation [16,17,18,19,20]. In contrast, NOTCH inactivating mutations predominantly occur in the epidermal growth factor (EGF)-like repeats of the extracellular portion of the receptors. These mutations, including point mutations, focal deletions, and nonsense and missense mutations, are predicted to prevent ligand binding or generate a truncated protein and are often associated with downregulated expression of NOTCH target genes [21,22,23,24,25,26].

Historically, NOTCH signaling in cancer has been extensively studied in the context of T-ALL, where NOTCH acts as an oncogene and NOTCH-activating mutations are present in more than 50% of patients [17]. Different laboratories have identified translocations or mutation clusters within the *NOTCH1* receptor gene that significantly favor tumor progression by causing a ligand-independent constitutive activation of the pathway [27]. Evidence that NOTCH can also promote tumor growth in solid tumors comes, for example, from breast cancer. It has been demonstrated that integration of the mouse mammary tumor virus (MMTV) causes rearrangement and activation of a particular locus containing the *Notch4* sequence and this ultimately results in cancer development [28,29]. Recurrent gene rearrangements in *NOTCH1* and *NOTCH2*, inducing NOTCH pathway activation, have also been identified in human estrogen receptor (ER)-negative and triple-negative breast cancer samples and cell lines [19]. In T-ALL and breast cancers, evidence points to c-Myc as an important effector of the tumor-promoting function of NOTCH [17,19,30]. However, NOTCH could also cooperate with known “cancer drivers” including breast cancer gene 1 (BRCA1), whose loss-of-function is responsible for the majority of breast cancers [31]. Miao and colleagues (2020) have recently suggested that NOTCH1 activation can regulate the cell cycle and attenuate BRCA1 deficiency-induced G2/M blockade and genomic instability, thus providing tumor cells with a survival advantage [31]. Moreover, it has been proposed that NOTCH signaling could promote epithelial-to-mesenchymal transition (EMT), enhancing tumor cell aggressiveness and metastatic potential [31,32].

The NOTCH pathway can also be tumor-suppressive in other contexts. The first evidence for a tumor suppressor role of NOTCH came from studies in mouse and human keratinocytes [33,34]. Later, NOTCH-inactivating mutations were identified in head and neck squamous cell carcinoma (HNSCC) [21,22] and small cell lung cancer (SCLC) [35,36,37]. In 2011, two independent whole-genome sequencing studies reported NOTCH-inactivating mutations in two different cohorts of patients with HNSCC [21,22]. These findings were further supported by a more recent and comprehensive genomic characterization of HNSCC that found *NOTCH* mutations in approximately 20% of the patients [38]. In SCLC, a highly aggressive and therapy-resistant lung cancer characterized by the expression of neuroendocrine (NE) markers, a recent in vivo clonal analysis demonstrated that a rare population of pulmonary NE stem cells could be induced to reactivate and differentiate upon injury and that this process is regulated by NOTCH. However, when NOTCH signaling is inhibited, the differentiation program is blocked and NE cells remain in a highly self-renewing state that is prone to transformation [39]. NOTCH-inactivating mutations correlating with a poorer patient prognosis have also been found in approximately 40% of patients with bladder cancer. Intriguingly, half of those patients did not carry other concomitant mutations in well-known oncogenic drivers including FGFR3 or RAS, suggesting a prominent role for NOTCH signaling in tumor initiation [23]. Moreover, complete or partial loss of chromosome 9, where the *NOTCH1* gene is located (9q34.3), is a common chromosomal aberration in bladder carcinoma [40]. In these tumors, NOTCH acts as a suppressor of cell proliferation by upregulating multiple members of the dual-specific phosphatase (DUSP) family, which inhibit Extracellular signal-Regulated Kinase 1/2 (ERK1/2) phosphorylation. As a consequence, *NOTCH* mutant tumor cells show increased ERK1/2 phosphorylation that can be reverted by NOTCH activation [23].

Intriguingly, there is evidence that NOTCH signaling can play both tumor-promoting and tumor-suppressive roles, even within the same organ. For instance, although the growth of HNSCC is largely driven by NOTCH inactivation [41], occasional Notch gain-of-function mutations have been reported in oral squamous cell carcinoma (OSCC) [42]. In the hematopoietic system, while an oncogenic role of NOTCH has been described in both acute (T-ALL) and chronic (CLL) forms of lymphocytic leukemia [17,18], a tumor-suppressive role has been proposed in chronic myelomonocytic leukemia (CMML) [43] and also suggested in acute myeloid leukemia (AML) [44]. Such dualism has been linked to the function of NOTCH in the regulation of cell fate choices during immune cell development. Multiple in vitro and in vivo studies have demonstrated that NOTCH favors T cell over B cell commitment and myeloid differentiation [45,46,47]. Consequently, NOTCH gain-of-function mutations lead to a rapid and abnormal expansion of T cells at the expense of other cell lineages, whereas NOTCH inactivation, particularly in the stromal compartment, causes an increase in myeloid progenitors and granulocyte/macrophage descendants, resulting in myeloid hyperplasia and myeloproliferative-like disease [43,48,49,50,51]. Hence, depending on the cell type, NOTCH signaling can play opposite roles in the development of hematological malignancies. A dual role for NOTCH signaling is also evident in some solid tumors, including lung cancer. The most prevalent form of lung tumors is non-small-cell lung cancer (NSCLC), a heterogeneous group of neoplasms that includes lung adenocarcinoma and squamous cell lung carcinoma in which NOTCH signaling activity has been suggested to promote and suppress tumor growth, respectively [14,37,52,53,54]. However, perhaps the most emblematic example of the fascinating complexity of how the NOTCH pathway can orchestrate tumor development is given by SCLC, an infrequent but very aggressive subtype of lung cancer. Lim and colleagues (2017) proposed that NOTCH signaling can simultaneously be oncogenic and tumor-suppressive in different cell subpopulations of an individual tumor, although intratumoral heterogeneity generated by NOTCH activity promotes overall SCLC growth [55]. The authors described the presence of two “symbiotic” cell types: slowly-proliferating and chemoresistant non-NE cells and actively dividing NE cells. NOTCH activation triggers a non-NE fate switch that slows tumor growth but also gives rise to non-NE cells that sustain NE cell expansion by providing trophic support. Therefore, while NOTCH activation delays initial tumor progression, it can provide a survival advantage after chemotherapy and fuel rapid tumor relapse [55].

## 2. NOTCH Signaling in Neural Stem/Progenitor Cells and Glioma Formation

Gliomas account for approximately 30% of all brain tumors and around 80% of malignant brain tumors [56]. They can be generically divided into diffuse low- and intermediate-grade II–III gliomas (low-grade gliomas, LGGs) and the most aggressive grade IV gliomas or glioblastoma multiforme (GBM). Although patients with LGGs have a more favorable prognosis, the diffuse infiltrative nature of LGGs makes a complete neurosurgical resection almost impossible, often resulting in recurrence and progression towards higher-grade gliomas [24]. Indeed, around 20% of GBMs are secondary tumors arising from preexisting LGGs [26]. Recent advances in omics techniques have allowed for the integration of clinical and histopathological data with a deep characterization of the genetic, epigenetic, expression, and metabolic profiles of gliomas [57]. The scenario that has emerged is that of a heterogeneous group of glioma subtypes, each characterized by different survival rates and responses to treatments. The importance of these findings has encouraged the World Health Organization to include molecular parameters together with the classical histological markers in the diagnosis of gliomas [58]. Given the broad heterogeneity within gliomas and the highly context-dependent roles of NOTCH signaling in different cancers even within the same tumor type, it is perhaps not surprising that the functions of NOTCH in glioma still remain controversial.

NOTCH activity is fundamental to maintain neural stem cell (NSC) and progenitor identity and to regulate cell fate decisions in the developing and adult brain [4]. Since uncontrolled proliferation and impaired differentiation of NSCs and glial progenitors may lead to glioma formation [59,60], understanding how these processes are regulated would be important to target cancer-initiating cells. After formation of the central nervous system is completed, NSCs persist within two restricted regions of the mammalian brain, the subgranular zone of the hippocampal dentate gyrus (DG) and the subventricular zone (SVZ) of the lateral ventricles [61,62]. Most adult NSCs are quiescent and only a proportion of them divide at any given time to self-renew and generate intermediate progenitors (IPs). IPs, in contrast, actively proliferate and differentiate mainly into neuroblasts, although glial cells can also be generated in the adult NSC niches [63,64,65]. The entire process is finely tuned by a variety of different mechanisms that ensure neuron generation and, at the same time, maintenance of a stem cell pool. Within the neurogenic lineage, the canonical RBPJ-mediated NOTCH pathway and the NOTCH target gene *Hes5* are restricted to NSCs [66,67,68,69]. While there is evidence for adult stem and progenitor heterogeneity, NOTCH-dependence seems to be a common feature among NSC populations [62]. Indeed, both quiescent and actively proliferating NSCs express *Hes5*, indicating NOTCH pathway activation [70,71,72], and simultaneous *Notch1* and *Notch2* or *Rbpj* gene deletion in mice causes NSC proliferation, differentiation, and exhaustion [67,68,73]. Interestingly, however, and in contrast to global NOTCH pathway inhibition, blocking the function of individual NOTCH receptors differentially affects active versus quiescent NSC populations, which seem to preferentially rely on NOTCH1 and NOTCH2/3, respectively [73,74,75,76,77]. Recent data from our lab suggest that NOTCH2 can promote NSC quiescence by inducing the expression of ID4 [77]. ID4 and NOTCH signaling synergize to inhibit excessive accumulation of the proneural factor ASCL1 [77,78], which stimulates NSC proliferation and differentiation [79,80]. While NOTCH target genes of the HES/HEY family repress *ASCL1* gene transcription [78], ID4 also facilitates the degradation of ASCL1 protein [81].

NOTCH signaling also regulates both the astrocyte and oligodendrocyte lineages. During embryonic development, NOTCH favors the gliogenic switch of radial glial cells by driving the expression of glial cell markers GFAP [82], BLBP [83], and NFIA [84]. The induction of an astrogliogenic program also requires NOTCH signaling after ischemia in the adult brain [85]. NOTCH signaling remains active in differentiated astrocytes but is modulated in response to injury. In striatal astrocytes, NOTCH signaling is reduced after stroke, resulting in ectopic glial proliferation and production of new neurons in non-neurogenic regions [86]. Reports indicate that NOTCH signaling also modulates the fate of oligodendrocyte progenitor cells (OPCs), another important cell of origin of glioma [60]. Jagged1-induced canonical NOTCH signaling restricts oligodendrocyte maturation and maintains OPCs during brain development and remyelination [87,88,89]. However, F3/Contactin can act as a non-canonical NOTCH ligand to promote OPC differentiation in a developmental stage specific manner [90].

Altogether, it is clear that NOTCH signaling plays a central role during homeostasis and injury response in NSCs and glial progenitors, both of which are potential cells-of-origin of brain tumors [60,91,92,93,94]. In line with this, NOTCH ligands, receptors, and downstream targets are expressed in several types of brain tumor [95,96,97,98,99,100] and varying levels of NOTCH activity can contribute to intra-tumor heterogeneity by promoting stem cell character in subpopulations of glioma cells [101,102]. Clearly, NOTCH signaling potentially regulates multiple steps of gliomagenesis, including tumor initiation, progression, and recurrence. Yet, a consensus on the role of NOTCH in glioma development is still missing.

## 3. NOTCH as an Oncogene in Glioma

**Figure 1:**
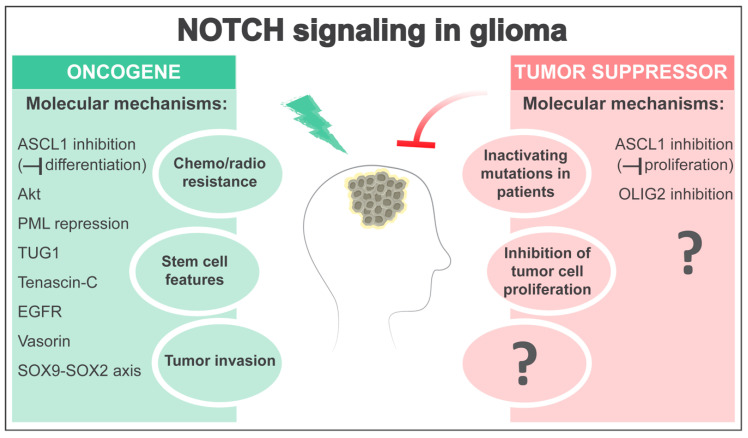
The NOTCH pathway can act either as an oncogene or as a tumor suppressor in glioma, depending on the context. On the one hand, NOTCH signaling activity in subpopulations of glioma cells can enhance stem cell features, promote resistance to radio- and chemo-therapies, and favor tumor development by activating oncogenic pathways (e.g., PI3K/Akt) or inhibiting tumor suppressors (e.g., PML). NOTCH can also regulate long non-coding RNAs such as TUG1 to maintain stemness and suppress differentiation. Moreover, NOTCH signaling can establish positive feedback loops with Tenascin-C, EGFR, and a SOX9-SOX2 axis. Finally, NOTCH can modulate interactions between glioma stem cells (GSCs) and their niche in different locations within the tumor mass, including in hypoxic regions (through Vasorin) and at the invasive front (through SOX9-SOX2), thereby promoting stem cell features and invasive potential. On the other hand, NOTCH-inactivating mutations and low expression levels of canonical NOTCH target genes have been identified in patients with glioma subtypes, and genetic NOTCH inhibition accelerates glioma formation in glioma mouse models, pointing to a tumor-suppressive role of NOTCH in glioma similar to in epithelial cancers. Although the molecular mechanisms driving a tumor-suppressive role of NOTCH signaling in glioma are still largely unknown, NOTCH can inhibit tumor cell proliferation and glioma growth by suppressing ASCL1 and OLIG2 expression. Interestingly, NOTCH-mediated suppression of ASCL1 can result in either oncogenic or tumor-suppressive effects by inhibiting differentiation or proliferation of glioma cells, respectively.

Evidence indicates that NOTCH signaling can promote glioma aggressiveness in some contexts (Figure 1). The presence of self-renewing glioma stem cells (GSCs) that have increased DNA repair capacity and expression of ATP-binding cassette (ABC) multidrug transporters, and that differentiate into less-tumorigenic cancer cells that form the tumor bulk, is one phenomenon that can confer therapy resistance in glioma [101,103,104,105,106]. Reminiscent of its role in healthy NSCs, NOTCH can facilitate stem cell character in brain tumors and has therefore been considered a promising target for the development of more effective glioma therapies. Data from both human tumors and murine glioma models suggest that NOTCH signaling is preferentially active in subpopulations of glioma cells [101,102,107]. In agreement with this, in vitro and xenotransplantation studies with glioma cell lines have indicated that CD133-positive GSCs are particularly sensitive to γ-secretase inhibitors (GSI) or *NOTCH1/2* knockdown compared to CD133-negative glioma cells [108,109]. Blocking NOTCH signaling or RBPJ reduced clonogenic potential in tumor-sphere assays and engraftment capacity in glioma xenograft models [108,109,110]. Conversely NICD overexpression, although not sufficient alone to induce brain tumors in the mouse brain [111,112], could increase cell survival due to radio-resistance and side population phenotype in glioma cells [101,108,109]. Combinatorial treatment of GSIs and radiation was more effective at inhibiting self-renewal than radiation alone [109,113], a synergistic effect that was partially mediated by NOTCH enhancing Akt and Stat3 phosphorylation [108,109]. NOTCH signaling could also promote a malignant phenotype in human glioma cell lines and xenograft models by repressing the expression of the promyelocytic leukemia protein (PML) tumor suppressor [114] and inducing the oncogenic long non-coding RNA TUG1 [115]. Reports have shown that knocking down the NOTCH ligands Jagged1 or Dll1 by RNA interference reduced survival and growth of tumor cells in multiple glioma cell lines [99,116] and high Jagged1 expression correlates with poor prognosis of glioma patients [116]. Interestingly, the extracellular matrix glycoprotein Tenascin-C and Jagged1 can reinforce each other’s expression, which could establish a feedback loop promoting tumor growth [116,117,118]. However, it is important to note that high levels of Jagged1 can also inhibit canonical NOTCH signaling in glioma cells, potentially through the activity of the Jagged1 intracellular domain [119].

In the adult SVZ neurogenic niche, NOTCH signaling plays a central role in maintaining the quiescent NSC pool [68,73], which is resistant to antimitotic treatment and can regenerate more actively proliferating progenitor cells [120,121]. Similarly, a relatively quiescent subset of chemotherapy-resistant glioma cells can propagate tumor growth after temozolomide administration in mouse models of GBM [104]. In line with this finding, a recent report demonstrated that pharmacological inhibition of receptor tyrosine kinases (RTK) prompts the emergence of slow-cycling and drug-tolerant persister cells in a subset of PDGFRA-amplified human glioma cell lines, and that an increase in NOTCH signaling activity allows transition to the persister state [122]. Although resistance to RTK inhibition could also develop independent of NOTCH in some cell clones [123], these data indicate that NOTCH can contribute to cell plasticity and drug tolerance in glioma by virtue of its stem cell-promoting activity. In this context, NOTCH inhibition could be exploited to release high expression of the proneural transcription factor ASCL1 in a subset of GSCs and induce their terminal neuronal differentiation [124,125].

Adult SVZ NSCs reside in a specialized vascular niche [126,127] that can foster stem cell character and repress differentiation through endothelial-derived factors that positively modulate NOTCH-dependent transcription [66,128]. In analogy to the SVZ niche, it has been proposed that GSCs reside in the proximity of blood vessels and are exposed to factors produced by endothelial cells [129]. Among these, nitric oxide has been shown to activate NOTCH signaling and promote stem-like character in PDGF-driven glioma [101]. Interestingly, NOTCH signaling is also augmented in hypoxic tumor regions by Vasorin-mediated stabilization of NICD, and hypoxia-induced expression of Vasorin promotes glioma aggressiveness [130]. Finally, there is evidence for dormant GSC populations residing at the invasive front of the tumor [131], where Jagged1 expressed by nerve fibers could facilitate NOTCH1^+^CD133^+^ glioma cell invasion of white matter tracts through a SOX9-SOX2-NOTCH1 feedback loop [132].

## 4. NOTCH as a Tumor Suppressor in Glioma

Surprisingly, recent data suggest that NOTCH signal inhibition may be an important molecular event in the formation of some forms of glioma (Figure 1). Genome-wide analyses in patients with LGG identified mutations in NOTCH signaling components in a significant proportion of isocitrate dehydrogenase (IDH) mutant tumors [24,25,26,133]. These mutations are particularly frequent in the genes encoding the NOTCH1 and NOTCH2 receptors but less common in NOTCH3/4, and occur at similar positions to those demonstrated experimentally to inactivate NOTCH1 function in epithelial cancers [24,25,26,133]. In addition, less frequent mutations in the *RBPJ* gene were detected in LGG samples and were mutually exclusive with *NOTCH1* mutations [134]. Accordingly, the expression of some NOTCH target genes is downregulated in *NOTCH/RBPJ* mutant gliomas, indicating pathway inactivation [24,26,134]. Expression of the proliferation marker MKI67 was found to be augmented in *NOTCH1/RBPJ* mutant brain tumors with low *HES/HEY* expression levels [134], and *NOTCH* mutations were a high-risk factor associated with shorter patient survival [135], suggesting that NOTCH signaling inhibition contributes to increased glioma aggressiveness. Consistent with this, frequent NOTCH pathway alterations were also detected when comparing progressed tumors with their corresponding lower-grade counterparts [26]. Although occasional *NOTCH1/2* mutations could also be detected in IDH mutant astrocytomas with *TP53* inactivation [25], genetic inactivation of NOTCH pathway components mainly occurs in oligodendrogliomas with co-deletion of chromosome arms 1p and 19q [24,25]. This is intriguing, as the genes for *NOTCH2*, *MIB2*, and the NOTCH targets *HES2-5* and *HEYL* are all located on chromosome arm 1p [136,137], lending support to the hypothesis that NOTCH signaling inhibition contributes to the initiation of tumors with oligodendroglial characteristics. Interestingly, genetic inhibition of NOTCH can promote OPC proliferation and expansion in mouse models [138], and excessive activation of quiescent OPC populations leads to malignant transformation [139].

In contrast to LGG, *NOTCH* mutations are very rare in GBM. However, it is worth noting that levels of NOTCH signaling activation substantially vary among GBM samples and subtypes [98,107], and hemizygous or homozygous deletions of the *1p36* locus, which could affect the *MIB2* and *HES2-5* genes, occur in a proportion of GBMs [140,141]. Although a number of genes have been proposed as *1p36* candidate tumor suppressors, it is unclear if NOTCH inhibition can contribute to cancer development after *1p36* loss.

Indirect evidence that NOTCH signaling could have tumor suppressor activity in glioma comes from studies on the proneural transcription factor ASCL1, whose expression is normally repressed by canonical NOTCH targets of the HES/HEY family in NSCs and GSCs [78,107,124,142]. Lineage tracing experiments in mice have shown that ASCL1^+^ neural progenitors can be cells of origin of GBM [59,91] and upregulation of ASCL1 and inhibition of NOTCH signaling characterize astrocytoma progression [143]. *ASCL1* expression is maintained in both xenografts from human proneural GBM samples and GBM mouse models, and ASCL1 can induce cell cycle genes and oncogenes, thereby promoting glioma cell proliferation in some contexts [122,144,145,146]. Accordingly, *ASCL1* knockdown or conditional gene knockout prolong survival of glioma-bearing mice [145,146]. In contrast, high expression of a curated NOTCH-signaling gene set, including HES/HEY transcriptional repressors, correlates with less proliferative glioma cell subpopulations with putative GSC character [122]. These data are in line with the role of NOTCH signaling in reducing *ASCL1* expression and preserving quiescence in NSCs [68,78,79,86,107].

More direct evidence that NOTCH signaling could restrict glioma formation in some contexts comes from studies taking advantage of genetic conditional gene deletion or overexpression approaches in vivo. NOTCH2 overexpression can inhibit glioma formation in mouse glioma models [107] and HEY2 overexpression can reduce the proliferation of murine and human glioma cells [147]. Conversely, ID2-mediated repression of HEY1 promotes NSC transformation, possibly by releasing the expression of OLIG2 [148], an oligodendroglial lineage determinant involved in glioma cell proliferation [149]. Simultaneous genetic deletion of *Notch1* and *Notch2* or *Rbpj* accelerates the growth of PDGF-driven GBMs in mice [107]. Moreover, inactivation of the *Rbpj* gene together with the tumor suppressor gene *Trp53* induces aggressive, de novo forebrain tumors with primitive neuroectodermal features [107]. These findings are in line with an unbiased in vivo CRISPR screen that recently identified NOTCH1 among potential tumor suppressors in GBM [150] and with the observation that high expression of specific NOTCH target genes positively correlates with a better prognosis in defined subsets of LGGs and GBMs in humans [107].

## 5. Open Questions and Perspectives

NOTCH receptors and their downstream targets are potential candidates for specific drug targeting, and various strategies to modulate NOTCH for cancer therapy are being actively pursued [151]. However, understanding the molecular basis of NOTCH oncogenic and tumor-suppressive functions is fundamental in order to develop effective strategies to therapeutically modulate NOTCH in glioma. Although clinical trials with GSIs in patients with glioma have been reported, positive effects have only been seen in one study [151,152]. Given the broad spectrum of gamma-secretase target proteins and the dose-limiting toxicities of GSIs, it would be interesting to test the effects of small molecules that directly target the NOTCH transcriptional activation complex in glioma [153,154,155]. Considering the extensive intertumoral heterogeneity in glioma, it is possible that NOTCH inhibition would be beneficial only in a proportion of molecularly selected patients in a personalized therapy. For instance, studies in vitro suggest that PTEN and TP53 status may affect sensitivity to GSIs in GBM [156,157]. NOTCH and EGF receptor pathways can potentiate each other in glioma cells [158,159,160] and, therefore, the oncogenic function of NOTCH signaling could be more apparent in primary GBMs of the classical subtype [161] than in GBMs with OPC-like proneural features [162] or IDH mutant LGGs [24,25]. The precise role of NOTCH in different forms of glioma requires further studies.

To some extent, the strength of NOTCH signaling might also explain the reported discrepancies in the oncogenic versus tumor-suppressive functions of NOTCH in the brain. NOTCH signaling is required to maintain a low and oscillatory *ASCL1* expression in order to promote NSC self-renewal [6,78]. However, sustained NOTCH signaling activation induces proliferative quiescence of NSCs [78]. Conversely, NOTCH inhibition induces activation of latent parenchymal progenitors [86] and NSC hyperproliferation before resulting in neuronal differentiation and NSC depletion [78]. Reminiscent of this, NOTCH inhibition could induce terminal differentiation of glioma cells in some contexts [124] but facilitate their proliferation in others [107]. NOTCH inhibition can contribute to the initiation of epithelial cancers by favoring the expansion of *NOTCH* mutant clones at the expense of wild-type cells [163]. Since analogous cell competition mechanisms play important roles during cancer progression [164], it would be interesting to determine if NOTCH-regulated competitive interactions occur between adjacent neural progenitors and if *NOTCH* mutations can contribute to glioma formation in this context. Reversible NOTCH signal activation likely plays a role in regulating glioma cell behavior during tumor development, but NOTCH-independent clones may arise under prolonged drug selection pressure [123]. Hence, it will also be important to address if the role of NOTCH in glioma varies on the basis of the stage of disease progression, as suggested for SCLC [55].

Finally, indication for a tumor-suppressive activity of the NOTCH pathway in glioma predominantly comes from an in vivo immunocompetent setting, and a recent CRISPR screen identified frequent co-mutation of the NOTCH1 receptor and B2m, an essential component of the MHC-I antigen presentation complex [150]. Interestingly, findings indicate that the immune response to cancer in the brain is shaped by the cancer type [165,166]. Whether NOTCH activity in tumor cells can regulate interactions with the glioma microenvironment and immune evasion remains unexplored.

## 6. Concluding Remarks

NOTCH signaling can act as an oncogene or a tumor suppressor, depending on the context [1]. Although it is clear that NOTCH plays central roles in glioma, its precise function has remained puzzling. The data reviewed here support the hypothesis of a dual role of the NOTCH pathway as an oncogene and a tumor suppressor in glioma (Figure 1), similar to what has been suggested in some other malignancies. Human gliomas comprise multiple disease subtypes that differ at the genetic, epigenetic, and transcriptional levels, and this intertumoral heterogeneity could be one critical factor underlying the observed discrepancies in NOTCH function. In addition, differences in the outcome of NOTCH modulation likely relate to the stage of disease progression, crosstalk with other signaling pathways, and intratumoral (stem) cell heterogeneity. While diverse molecular bases of NOTCH oncogenic function in glioma have been addressed in previous studies, mechanistic data on the NOTCH tumor-suppressive activity in brain tumor subtypes are still lacking. It is also unclear if NOTCH-regulated cell competition mechanisms are in place during glioma initiation and progression. Finally, whether NOTCH activity in glioma cells regulates the crosstalk with the tumor microenvironment and immune cells in particular remains unexplored. Clearly, the multiple tasks of NOTCH signaling in glioma deserve further scrutiny.
